# Comparative transcriptomics analysis of contrasting varieties of *Eucalyptus camaldulensis* reveals wind resistance genes

**DOI:** 10.7717/peerj.12954

**Published:** 2022-02-24

**Authors:** Xiuhua Shang, Peijian Zhang, Guo Liu, Ni Zhan, Zhihua Wu

**Affiliations:** China Eucalypt Research Centre, Chinese Academy of Forestry, Zhanjiang, Guangdong, China

**Keywords:** *Eucalyptus camaldulensis*, Wind stress, Transcriptional sequencing, Wind resistance genes

## Abstract

**Background:**

Wind, an important abiotic stress factor, affects forests in coastal areas, causes tree damage and timber loss.

**Methods:**

Two genotypes of *Eucalyptus camaldulensis-*strong wind-resistant CA5 and weak wind-resistant C037 were used for RNA-seq analysis to screen for candidate wind-resistance genes and transcription factors (TFs) by comparing the transcriptome analysis of the two varieties in response to wind stress.

**Results:**

It showed that 7061 differentially expressed unigenes could be annotated including 4,110 up-regulated unigenes and 2,951 down-regulated unigenes. Gene Ontology (GO) analysis revealed that six cellulose pathways were involved in response to wind stress. The unigenes in phenylpropanoid biosynthesis, phenylalanine metabolism, and flavonoid biosynthesis pathways were found to be differentially expressed based on Kyoto Encyclopedia of Genes and Genomes (KEGG) analysis. Moreover, 37 differentially expressed genes were functionally annotated to be involved in the secondary metabolism of phenylalanine (ko00940). Seventy-eight TFs related to the regulating cellulose and lignin synthesis were expressed differently from the various treatments. The expressions of C3H, POX, MYB, NAC, Gene008307, and Gene011799 were significantly upregulated in CA5. Overall, the main response of *Eucalyptus* to wind stress was associated with cell wall biosynthesis; key genes of cellulose and lignin biosynthesis pathways and related TFs were involved in the tree response to wind stress.

## Introduction

The wind is an important abiotic stress factor that causes forest damage, followed by other factors such as fires, pests, and diseases (Défossez et al., 2015). Upon reaching their limits of withstanding wind pressure, specific parts of trees cannot further resist the wind load, which results in trunk bending, crown and trunk breaking (windbreak), uprooting, and other injuries (Gardiner et al., 2008; Peltola et al., 2000). Wind damage has caused huge economic losses in the forestry sector. In Europe, wind damage accounts for 50% of the forest loss. The wind-induced damage to forests is anticipated to increase, consequently affecting the forest economy (Défossez et al., 2015). For example, the loss of a considerable amount of timber in Finland was witnessed due to storms (Zubizarreta-Gerendiain, Pukkala & Peltola, 2016; Gregow et al., 2008), and the planting area of Eucalyptus in Brazil decreased sharply due to the wind-related disasters (Zanuncio et al., 2017). In addition, wind damage hampers the structure and function maintenance of forest ecosystems in coastal areas; besides, it impacts the composition, succession, and function of forest ecosystems (Imbert & Portecop, 2008; Coomes et al., 2018). Based on the Amazon region as an example, the annual carbon storage loss caused by wind damage is 1.3 pg, while the annual carbon storage reduction caused by logging is merely 0.2 pg (Espírito-Santo et al., 2014); therefore, wind damage is the main factor causing the sharp reduction of forest carbon storage (Jackson et al., 2019). Moreover, wind damage is one of the main environmental factors that significantly affect plant growth, development, and distribution (Xu et al., 2017). Wind inhibits the water transport capacity of plants, resulting in water deficiency in leaves, and limited photosynthesis. Long-term winds significantly influence the growth and wood properties of trees. Strong winds, such as typhoons, not only shake trees but also affect their internal structure, which adversely impacts water regulation and photosynthetic physiology (James, Haritos & Ades, 2006; Ennos, 1997; Gardiner, Berry & Moulia, 2016). Additionally, trees shed leaves, tend to incline, and are broken and/or uprooted owing to strong typhoons.

At present, research regarding wind resistance of trees has been mainly focused on coniferous species such as *Acer pseudoplatanus* (Zubizarreta-Gerendiain, Pukkala & Peltola, 2016; Duperat, Gardiner & Ruel, 2021), Quercus robur (Jackson et al., 2019), *Maritime Pine* (Dupont et al., 2018), *Rubber* (Edzang et al., 2020; Wu et al., 2012), *Eucalyptus* (Zanuncio et al., 2017; Zanuncio et al., 2019; Shang et al., 2017), and coastal protective tree species such as *Casuarina equisetifolia* and *Acacia* (Xu et al., 2014; Wu et al., 2010). Of these, *Eucalyptus* is an important plantation tree species with high economic benefits in China. The planting area of *Eucalyptus* in China has reached 5.46 million hm^2^ with an annual output of more than 45 million m^3^ of wood, making an important contribution to wood production (Arnold et al., 2013; Xie et al., 2017). Research on wind resistance of *Eucalyptus* has been mainly focused on their growth and wood properties after typhoons. For example, Zanuncio et al. (2017) measured the wood properties of *E. grandis* ×* E. urophylla* to evaluate the resistance of *Eucalyptus* clones to wind damage. Luo et al. (2009) studied the genetic variation in growth and wind resistance of 2-year-old *Eucalyptus* hybrids. Shang et al. (2017) studied the effects on growth and wood properties with respect to wind resistance in 50 *E. camaldulensis* individuals. The results indicated that the wind damage index heritability was high and close to the heritability of fibre length. The heritability of individual trees and families was 0.516 and 0.524, respectively (Shang et al., 2017). Some studies based on field investigation and statistics were conducted in controlled conditions to evaluate the relationship between the wind damage index and individual tree traits to determine the factors influencing tree resistance to wind. However, resistance against winds in standing trees in fields can be attributed to many factors. Therefore, by exclusively using the conventional method including regression analysis of wind damage and tree traits, it is impossible to understand the key factors of wind resistance in trees and to elucidate the underlying mechanism. Abundant genetic resources with respect to wind resistance in *Eucalyptus* species are available (Luo et al., 2009; Shang et al., 2017), and it is important and urgent to utilize their wind resistance genes and develop new breeding-based varieties.

Plants can respond to external stimuli such as wind in terms of physiology and growth, which enable plants to withstand further wind challenges (Braam et al., 1997). Some studies have shown that various signalling molecules and phytohormones, including intracellular calcium, ethylene, abscisic acid, auxin, and reactive oxygen species have been implicated in reactions exhibited by physiological and morphological transformations (Nicoll et al., 1995). However, to understand the mechanical signals, signalling pathways and physiological response of plants to mechanical stimuli, further research is warranted (Telewski & Pruyn, 1998; Chehab, Eich & Braam, 2009). The plant cell wall is composed of cellulose, hemicellulose, lignin, polysaccharides, and proteins, which form a strong network of filaments, providing mechanical support for cells, tissues, and the whole plant (Gilbert, 2010). Cellulose, as the main component of the cell wall, significantly promotes maintenance of the mechanical strength of the stem (Xiang et al., 2010). Studies on lodging resistance in wheat, rice, corn, and other crops have found that an increase in the cellulose content can significantly improve the mechanical strength of the stem and enhance its compressive capacity; varieties with high cellulose content have strong lodging resistance (Yang et al., 2009; Huang et al., 2014). Additionally, lignin is a secondary metabolite that can determine the strength of the cell wall and lodging resistance of the stem; it plays a key role in maintaining the mechanical strength of the stem (Turner & Somerville, 1997; Lewis & Yamamoto, 1990). Studies on lodging resistance of oat (Welton, 1928), wheat (Tripathi & Sayre, 2003), rape (Liu & Guan, 2008), and rice (Ookawa & Ishihara, 1993) have shown that stems with high lignin content have high mechanical strength and strong lodging resistance, and the lignin content is significantly positively correlated with the lodging resistance of crops (Thompson, 1963; Zuber & Grogan, 1961). Our previous research found that Eucalyptus strains with high cellulose and lignin content have strong wind resistance. Combined with the lodging research in crops, it can be inferred that the content of cellulose and lignin in trees has an important impact on their wind resistance. Luo et al. (2009) studied that the wind resistance in 2-year Eucalypt hybrids was only controlled by additive genes, and the wind resistance of hybrid offspring was only determined by the wind resistance characteristics of parents. The interaction between parents had no significant effect on the wind resistance of offspring. The wind resistance index of Eucalyptus hybrids was controlled by additive genes and the heritability ranged from 0.10 to 0.11. The heritability of wind resistance index of offspring with *Eucalyptus urophylla* as a female parent was higher than that of offspring with *Eucalyptus tenuifolia* as a female parent. The heritability of wind resistance index of 1-year-old *Eucalyptus camaldulensis* was 0.2, and that of 4-year-old *Eucalyptus camaldulensis* is 0.52. The heritability of wind resistance index was different among different Eucalyptus varieties at different ages, indicating that the smaller the tree, the more susceptible it was to environmental effects. The heritability of fibre length, fibre width and wood density were higher, indicating that these traits were less affected by environmental conditions (Shang et al., 2017). Genetic improvement of wind resistance is an important way to deal with wind damage. Due to the long generation cycle and high genetic heterozygosity of trees, and most of the target improved traits are quantitative traits jointly controlled by multiple gene loci, the genetic improvement process of tree wind resistance is greatly limited by conventional breeding. So far, no wind resistance genes have been reported in forest trees, and the key genes for the synthesis and accumulation of *Eucalyptus* cellulose and lignin in the process of wind resistance are not understood. Therefore, in this study, two genotypes–strong wind-resistant CA5 and weak wind-resistant C037, were selected from *E. camaldulensis* families based on our previous research outcome; their cellulose and lignin contents were determined, and wind stress treatment was provided based on the tree-pulling test (Fredericksen, Hedden & Williams, 1993; Sani et al., 2012). The key candidate genes related to wind resistance in *Eucalyptus* were identified by high-throughput transcriptome sequencing (RNA-seq), and the results of the transcriptome sequencing were verified by conducting quantitative PCR analysis to provide a technical reference for breeding and efficient utilisation of wind-resistant varieties.

## Materials & Methods

### Plant material

For the trial, *E. camaldulensis* was planted in August 2012 in the South China Experiment Nursery, which is located in Suixi County, Guangdong Province. It was built for seed breeding from provenance forests of Australia and India, including 12 populations (provenances), 115 families, and more than 1700 individuals. The trial site and establishment have been described previously by Luo et al. (2014). In this study, the experimental materials were 7-year-old standing trees of two *E. camaldulensis* genotypes, CA5 (strong wind resistance) and C037 (weak wind resistance), whose wind resistance was measured and estimated based on wind damage caused by several typhoons. In September 2019, wind simulation experiments using tree-pulling tests were conducted in the field. The specific method involved applying the pulling force to the trunk at a tree height of 4 m by using a winch and cable to tilt the standing tree to 30° vertical to the trunk; three trees were selected and tested for each genotype. The immature xylem of the standing tree at breast height was taken at each of two time points, 0 h (before treatment) and 24 h (after treatment), and the wind-resistant CA5 had three biological replicates. Due to a trunk breakage of C037 in the test process, two biological replicates were considered. For simplicity, the samples of two genotypes extracted before and after treatment were named as C037_0 h and C037_24 h, and CA5_0 h and CA5_24 h, respectively, and then used for transcriptome sequencing.

### Determination of wood traits

Fibre length and fibre width (FW) were determined by LDA 02 Hi-Res Fiber Quality Analyzer(OPTEST, Canada). Bending strength referred to a method of testing in bending strength of wood (GB/T1936.1-2009 of PRC national standards), bending elastic modulus followed by the method for determination of the modulus of elasticity in static bending of wood (GB/T19362-2009 of PRC national standards). Strength of structural timber parallel to grain followed by the method of testing in shearing strength parallel to the grain of the wood(GB/T 1937-2009 of PRC national standards). Compressive strength parallel to grain was a method used to test the compressive strength parallel to the grain of the wood (GB/T 1935-2009 of PRC national standards). Browning (1967) method was used for the determination of *α*-cellulose content and holocellulose content, and Huntley et al. (2003) method for the determination of lignin content.

### Total RNA extraction and cDNA library construction

Total RNA was extracted using the RNAprep Pure Plant Kit (TIANGEN, Beijing, China), and the concentration and purity of the total RNA samples were detected using a NanoDrop 2000 (Thermo Scientific, Wilmington, DE, USA). The total RNA integrity of 28s/18s and RNA integrity number (RIN) values were detected by Agilent 2100 Bioanalyzer. Next, the mRNA was purified from total RNA with Poly-T oligomagnetic beads. The first-strand cDNA was synthesized with random hexamer primers and M-MuLV reverse transcriptase. The second cDNA strand was synthesised by DNA Polymerase I and RNase H. AMPure XP system (Beckman Coulter, Beverly, MA, USA) was used to purify the library fragment. Phusion High-Fidelity DNA polymerase, Universal PCR primer and Index (X) Primer were used for PCR. Finally, the PCR products were purified by the AMPure XP system, and the library quality was evaluated by Agilent Bioanalyzer 2100 system.

### Transcriptome sequencing and functional annotation

High-throughput sequencing was performed on an Illumina HiSeq6000 platform with three technical replicates. The raw reads obtained by transcriptome sequencing were filtered to obtain clean reads by removing reads containing adapters, reads containing poly-N, and low-quality reads from raw data. Meanwhile, Q20, Q30, GC content and sequence repetition level of clean data were calculated. The TopHAT software (Trapnell, Pachter & Salzberg, 2005) was used to compare the sequence of clean reads with the transcriptome sequence of the third generation of *E. camaldulensis*, and accurate position information of the reference genome was obtained. There were 7 databases for gene annotation: NR (NCBI non-redundant protein sequences), NT (NCBI non-redundant nucleotide), Pfam (Protein family), KOG/COG (Clusters of Orthologous Groups of proteins), Swiss-Prot (a manually annotated and reviewed protein sequence database), KO (KEGG homologous database), and GO (Gene Ontology). The personalised analysis platform and tools of the BMK cloud platform were used for in house analysis of transcriptome data and annotation of TFs.

### Single-nucleotide polymorphism (SNP) mining

Using software SOAPsnp (http://soap.genomics.org.cn/soapsnp.html) to get all of the unigene sequences SNP detection. The genes of different SNPs in CO37 and CA5 were mapped to the GO annotation file of *E. camaldulensis* genome, and the Go functional classification statistics and KEGG metabolic pathway analysis were performed for all genes.

### Analysis of differentially expressed genes (DEGs)

The CuffQuant and CuffNorm components of the CuffLinks software were used to quantify the transcription and expression levels of genes, and the fragment number per kilobase transcript (FPKM) was used as the index to measure the transcription or gene expression levels (Trapnell et al., 2010). For detecting DEGs, fold change ≥2 and FDR<0.01 were selected as screening criteria (Benjamini & Hochberg, 1995). After the DEGs were screened, BLAST analysis was performed between the DEG and NCBI databases (NR), Swiss-Prot, KEGG, COG, and KOG databases (E- value < 110-5). The DEGs were annotated with reference to the protein amino acid sequence information with high homology to the target gene. Goseq R packages based on Wallenius non- central hypergeometric distribution were used to classify the functions of DEGs in the GO database (Young et al., 2010). The KEGG signal pathway was enriched and analysed using the Kyoto Orthologics-based Annotation System2.0 (KEGG) signal pathway (Mao et al., 2005).

### Quantitative Real-time PCR (qRT-PCR) verification

The cDNA of the transcriptome backup sample was used as a template for gene amplification. Twelve differentially expressed genes related to cellulose and lignin were selected to verify the transcriptome data. Primers for qRT-PCR of related genes were designed using Primer 3.0 software, and *18S rRNA* was set as an internal reference gene, with three biological and three technical replicates. The primer information is included in Table S1. The cDNA of each sample was diluted and used as a template for the verification of differential genes by qRT-PCR. The qRT-PCR system is presented in Table S2. The qRT-PCR procedure was set according to the instructions corresponding to the fluorescent dye SYBR Green. qRT-PCR was performed using the analytikjena-qTOWER2.2 fluorescent quantitative PCR instrument. The procedure was as follows: 95 °C for 3 min, 95 °C for 10 s, and 58 °C for 30 s, for 39 cycles. The relative expression of genes was calculated using the 2^−ΔΔ*ct*^ method (Livak & Schmittgen, 2001).

## Results

### Differential analysis of wood quality index between C037 and CA5

The differences in the bending strength, bending elastic modulus, structural timber parallel to the grain, compressive strength parallel to the grain, lignin content cellulose and holocellulose content between CA5 and C037 were very significant (*P* ≤ 0.01) (Table 1). The differences in the fibre length, fibre width, *α*-cellulose content between CA5 and C037 were significant (*P* ≤ 0.05). According to the fibre and physical mechanics analysis, it is found that the fibre length is between 0.53–0.60 mm, the fibre width means was 25.40 µm in the range of 24.00–26.60 mm. Therefore, Nevertheless, there were significant differences in fibre length and fibre width among different families. Moreover, the cellulose and lignin content of CA5 was higher than that of C037. Further analysis results showed that lignin content varied from 19.40% to 26.10%, the average content of Lignin content was 22.32%, the minimum of HC was 76.91%, the maximum value was 82.84%, and the average HC was 79.83%. The range of holocellulose content was 77.00%-82.40%, with an average of 79.90%. The results showed that bending strength ranged from 58.00 MPa to 89.00 MPa with an average of 73.45 MPa, and bending elastic modulus ranged from 5056.00 MPa to 8470.00 MPa with an average of 6962.17MPa. The minimum value of compressive strength parallel to grain was 37.10 MPa, the maximum value was 53.10 MPa and the average value was 45.63 MPa. The minimum value of structural timber parallel to grain was 16.20 MPa, the maximum value was 31.30 MPa, the average value was 24.35 MPa. There are obvious differences between CA5 and C037. The heritability of structural timber parallel to the grain, Compressive strength parallel to the grain, Holocellulose content and *α*-cellulose content was more than 0.8, indicating that these characters were less affected by environmental factors. The heritability of fibre length and fibre width was less than 0.5, indicating that these two traits are susceptible to environmental factors.

**Table 1:** Basic descriptive statistics and ANOVA among between CA5 and C037.

Traits	Minimum	Maximum	Means	Skewness	Kurtosis	*P*-value	Heritability
Bending strength/MPa	58.00	89.00	73.45	−0.035	−3.061	0.000	0.647
Bending elastic modulus/MPa	5056.00	8470.00	6962.17	−0.288	−1.961	0.005	0.505
Structural timber parallel to grain/MPa	16.20	31.30	24.35	−0.122	−2.694	0.001	0.818
Compressive strength parallel to grain/MPa	37.10	53.10	45.63	−0.068	−2.329	0.004	0.851
Lignin content/%	19.40	26.10	22.32	0.158	−2.782	0.001	0.686
Holocellulose content/%	77.00	82.40	79.90	−0.161	−2.379	0.002	0.847
α-cellulose content/%	42.30	54.20	47.73	0.414	−1.237	0.020	0.814
Fiber length /mm	0.53	0.60	0.57	−0.416	−1.884	0.020	0.313
Fiber width /µm	24.00	26.60	25.40	−0.343	−1.841	0.012	0.439

### RNA sequencing data quality assessment

In total, 67.84 GB of clean data were obtained from 10 samples based on transcriptional analysis. For each sample, 5.89 GB of clean data were obtained, and the clean read number of the 10 samples was between 19697418 and 28879406. The variation in the number of clean bases was from 5893116176 to 8639188918. The GC content of the obtained data after filtration was between 51.01% and 52.05%. The Q20 and Q30 values were over 97% and over 94%, respectively. The average number of N (in the base) after filtration (Table S3). It indicates that the sequencing quality was high and accurate, and the raw RNA-seq data could be used for subsequent assembly. After a sequence similarity search against eight databases, we annotated 26588 different assembled unigenes (Table S4).

### SNP detection

SNP_S_ were screened from C037 and CA5 samples using SOAPsnp and their locations were determined. Statistics show (Table S5) that the number of SNP_S_ detected in the samples of non-wind-resistant strain C037 were 87018, 64805 and 57496, and the number of SNPS detected in the samples of wind-resistant strain CA5 was 67361, 63584 and 61089 respectively. Further analysis showed that C/T and A/G had the highest frequency of occurrence, which was more than 25%, while the frequency of other four single nucleotide variations A/C, G/T, C/G and A/T were less than 10%. Among the six variation types, the frequency of C/T was the highest, which may be because the methylated cytosine residues on CpG dinucleotide are easily spontaneously deaminated to form thymine. In order to further understand the information of SNP locus, the genotype of this locus and the mutated genotype of each individual were analyzed. According to the number of reads supporting this locus and the genotype of this locus obtained by GATK3 software, the genotype was different in the samples of wind resistance. According to the statistical results, the heterozygosity of C037 was 40.53%, and that of CA5 was 43.16%.

### GO classification of genes where SNP loci reside

**Figure 1:**
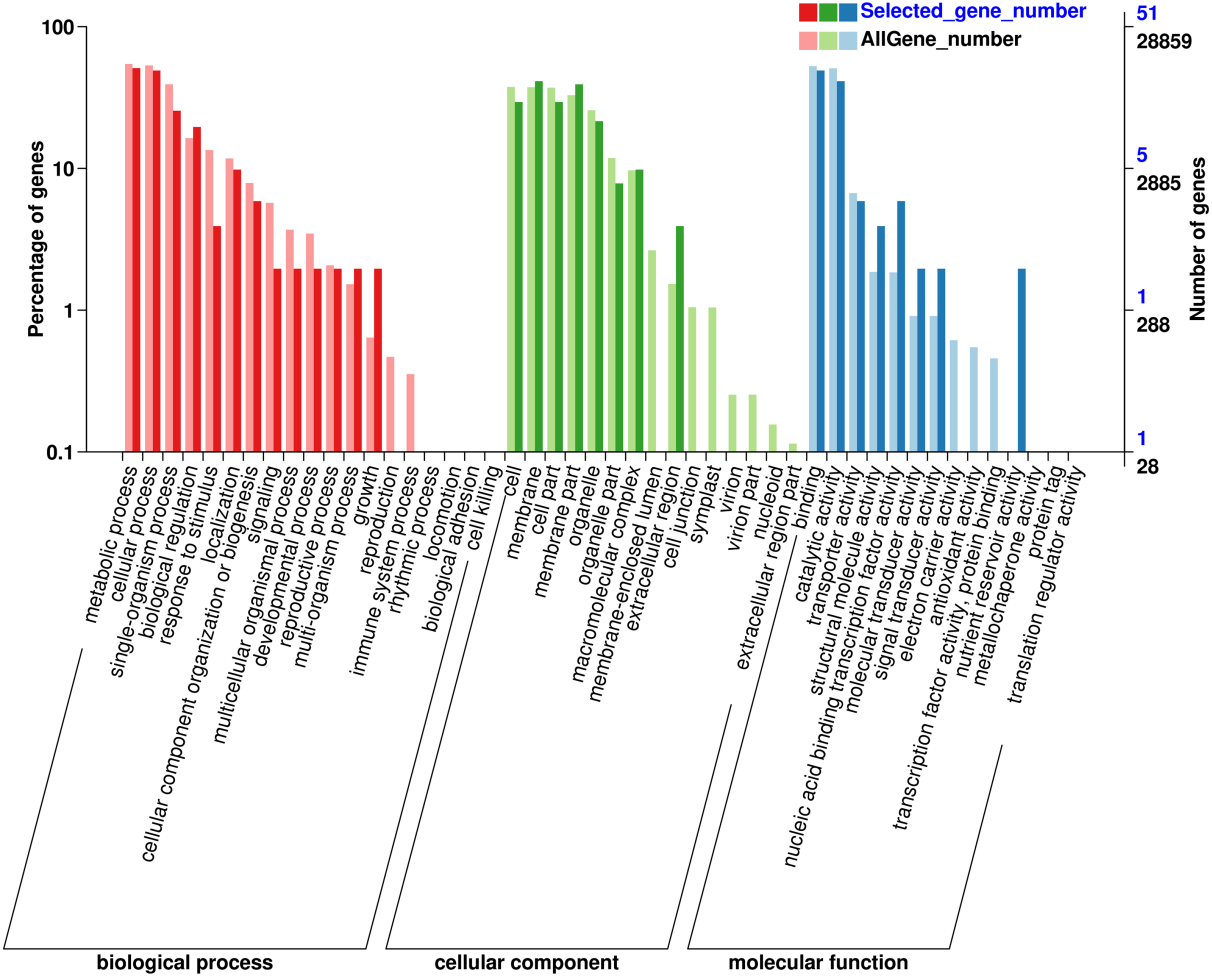
Gene Ontology (GO) analysis for genes with SNP.

In order to understand the function of the screened genes containing SNP, the GO annotation results were further classified. These genes were annotated into 29 functional regions in 3 categories (Fig. 1), including 90 genes in 13 functional regions of Biological process, 93 genes in 8 functional regions of Cellular Component and 57 genes in 8 Molecular functions. In biological processes, the most genes were involved in metabolic process (26), cellular process (25), single organization process (13) and biological regulation (10). Among the cell components, the genes involved in membrane (21), membrane part (20), cell and cell part (15) were the most. Among the molecular functions, the most genes were involved in binding (25) and catalytic activity (21). SNP containing genes was mainly related to the metabolic process of Eucalytus globulus.

### KEGG pathway analysis of SNP locus gene

**Figure 2:**
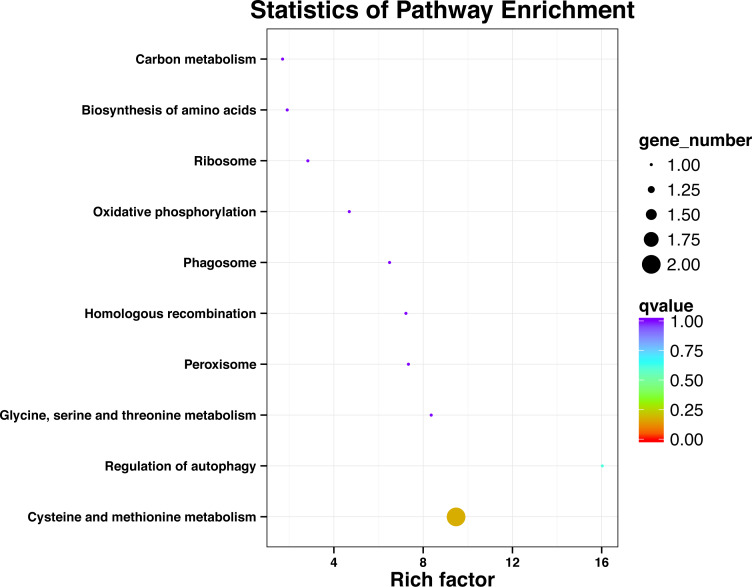
Significant enrichment pathways of KEGG for genes with SNP.

After processing the KEGG annotation results of 80 SNP containing differences unigene in the transcriptome data, it was found that 11 genes with known KEGG function have been annotated into 10 pathways (Fig. 2). Among them, cystaine and methionine metabolism had two unigenes, and the other pathways had one unigene. The results showed that both KEGG metabolic pathway analysis and GO classification were related to metabolism.

### Screening for DEGs

**Figure 3:**
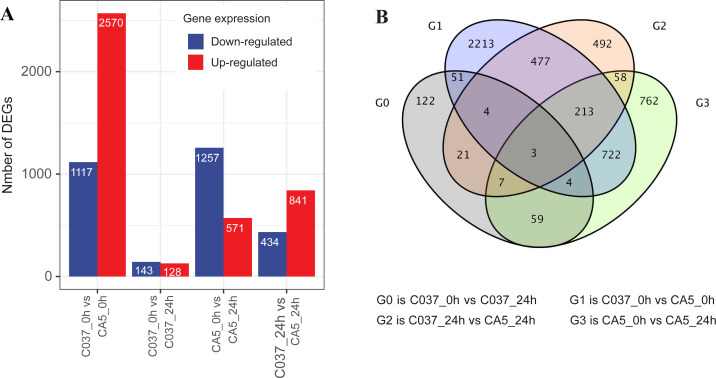
DEGs analysis of C037 and CA5 varieties under wind stress.

The DEGs were screened with a standard fold change (FC) and false discovery rate (FDR) with significant differences across the tested samples (Fig. 3). In total, 7061 DEGs were detected across the four samples, including 4110 upregulated genes and 2951 downregulated genes. We found 1828 DEGs in CA5 under wind stress (CA5_0 h *vs.* CA5_24h), including 571 upregulated genes and 1257 downregulated genes. However, only 271 DEGs were detected in C037 under wind stress (C037_0 h *vs.* C037_24h). More DEGs were identified in CA5 under wind stress compared to that in C037. There were 3687 DEGs in C037_0 h *vs.* CA5_0 h before wind stress, of which 2570 were upregulated and 1117 were downregulated. There were 1275 DEGs in C037_0 h *vs.* CA5_0 h after wind stress, of which 841 were upregulated and 434 were downregulated. A total of 697 genes overlapped between C037_0 h and CA5_0 h and C037_24 h *vs.* CA5_24 h, and only 73 genes overlapped between C037_0 h *vs.* C037_24 h *vs.* CA5_0 h *vs* CA5_24 h. The annotated DEGs of the different groups are shown in Table S6. Based on the generated volcano map, we can further visualize the significant difference in the expression levels between the CA5 and C037 samples (Table S7).

**Figure 4:**
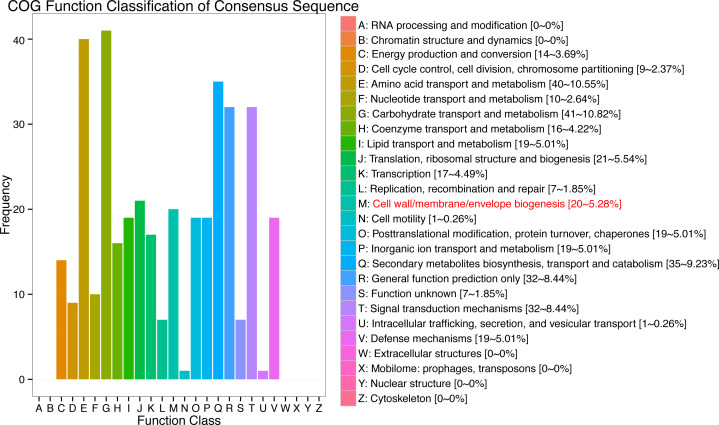
Cluster of orthologous groups of proteins (COG) functional classification for the obtained transcriptomic sequences of *Eucalyptus camaldulensis*. The unigenes have been classified into 20 COG categories (listed as C to V in the key on the right side of the figure). The *y*-axis indicates the number of unigenes in each COG category.

**Figure 5:**
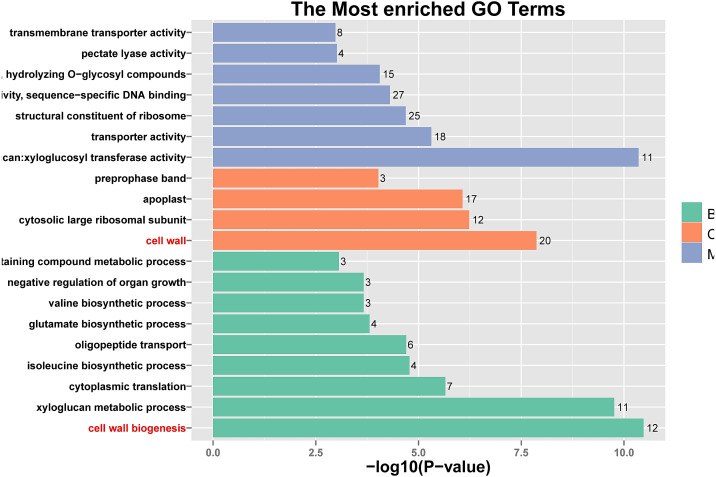
Histogram of Gene Ontology (GO) assignment for transcriptomic sequences of the 20 paths with the highest confidence *P*-value with 762 genes. The length of each bar (and numbers at the tail of each bar) indicates the unigenes’ *P*-values were transformed by -log10 in each sub-category.

In response to wind stress, 762 and 122 DEGs (Table S8) were exclusively detected in CA5 and C037, respectively; this indicates that wind resistance in *Eucalyptus* depends largely on the differential gene expression. After the clustering analysis of 7601 DEGs detected across these samples, the Clusters of Orthologous Groups of proteins (COG) and Kyoto Encyclopaedia of Genes and Genomes (KEGG) analyses were conducted for the 726 DEGs specific to CA5 in response to the wind stress. We found that cell wall and phenylpropanoid biosynthesis pathway genes potentially play a key role in response to wind stress (Figs. 4; 5). The genes related to cell wall synthesis, especially those involved in the lignin synthesis pathway, could be the key factors for variation in wind resistance across the different Eucalyptus varieties.

### GO enrichment analysis

GO enrichment analysis of the candidate wind-resistant DEGs in the four groups revealed that CA5 and C037 were significantly different in their biological processes, cell composition, and molecular function under wind resistance and control conditions. A total of 5,377 GO annotation entries were obtained corresponding to 28,859 genes. Upon further classification and enrichment of the three functional categories, the top five GO classifications with the most significant differences in gene expression at different time points and categories were obtained (Table 2). In terms of cellular components, the integral component of membrane (GO: 0006952) and extracellular region (GO: 0005576) were significantly enriched in all groups, and the integral component of the plasma membrane (GO: 0005887) was significantly enriched in C037_0 h *vs.* C037_24 h, CA5_0 h *vs* CA5_24 h, and C037_24 h *vs.* CA5_24 h. The number of biogenesis genes in CA5 was significantly higher than that in C037 cells. In terms of molecular function, the GO terms corresponding to ADP binding, ATP binding, and iron ion binding were significantly enriched across all the samples, and the protein serine/threonine kinase activity was found significantly enriched in C037_0 h *vs.* CA 50 h, C037_0 h *vs.* C037_24 h, and CA5_0 h *vs* CA5_24 h. In the biological processes category, terms for defence response and protein phosphorylation were significantly enriched across all the samples, and the processes of lignin biosynthesis were significantly enriched in C037_0 h *vs.* CA5_0 h, C037_24 h *vs.* CA5_24 h, and CA5_0 h *vs.* CA5_24 h after wind stress treatment. Compared with C037, CA5 exhibited two more processes of lignin biosynthesis and cell wall biogenesis under wind stress. These findings indicate that the wind resistance trait in wind-resistant genotypes may be related to cell wall biogenesis.

**Table 2:** GO enrichment of the DEGs between CA5 and C037.

Groups	Biological process	Molecular function	Cellular component
C037_0 h *vs* C037_24h	defence response (GO:0006952)	ADP binding (GO:0043531)	integral component of membrane (GO:0016021)
	protein phosphorylation (GO:0006468)	ATP binding (GO:0005524)	photosystem II oxygen evolving complex (GO:0009654)
	recognition of pollen (GO:0048544)	protein serine/threonine kinase activity (GO:0004674)	integral component of plasma membrane (GO:0005887)
	cellular transition metal ion homeostasis (GO:0046916)	monooxygenase activity (GO:0004497)	extracellular region (GO:0005576)
	double-strand break repair (GO:0006302)	iron ion binding (GO:0005506)	condensed chromosome (GO:0000793)
C037_0 h *vs* CA5_0h	defence response (GO:0006952)	ADP binding (GO:0043531)	integral component of membrane (GO:0016021)
	lignin biosynthetic process (GO:0009809)	protein serine/threonine kinase activity (GO:0004674)	microtubule (GO:0005874)
	plant-type secondary cell wall biogenesis (GO:0009834)	ATP binding (GO:0005524)	plasma membrane (GO:0005886)
	protein phosphorylation (GO:0006468)	microtubule binding (GO:0008017)	extracellular region (GO:0005576)
	xylan biosynthetic process (GO:0045492)	iron ion binding (GO:0005506)	kinesin complex (GO:0005871)
CA5_0 h *vs* CA5_24h	defence response (GO:0006952)	ADP binding (GO:0043531)	integral component of membrane (GO:0016021)
	protein phosphorylation (GO:0006468)	ATP binding (GO:0005524)	cell wall (GO:0005618)
	lignin biosynthetic process (GO:0009809)	protein serine/threonine kinase activity (GO:0004674)	Microtubule (GO:0005874)
	cell wall biogenesis (GO:0042546)	iron ion binding (GO:0005506)	extracellular region (GO:0005576)
	cellular transition metal ion homeostasis (GO:0046916)	monooxygenase activity (GO:0004497)	integral component of plasma membrane (GO:0005887)
CA5_24 h *vs* C037_24h	defence response (GO:0006952)	ADP binding (GO:0043531)	integral component of membrane (GO:0016021)
	protein phosphorylation (GO:0006468)	monooxygenase activity (GO:0004497)	extracellular region (GO:0005576)
	lignin biosynthetic process (GO:0009809)	iron ion binding (GO:0005506)	integral component of plasma membrane (GO:0005887)
	cellulose biosynthetic process (GO:0030244)	oxidoreductase activity (GO:0016705)	photosystem I (GO:0009522)
	recognition of pollen (GO:0048544)	ATP binding (GO:0005524)	photosystem II oxygen evolving complex (GO:0009654)

### KEGG-based pathways enrichment analysis of the related genes

**Figure 6:**
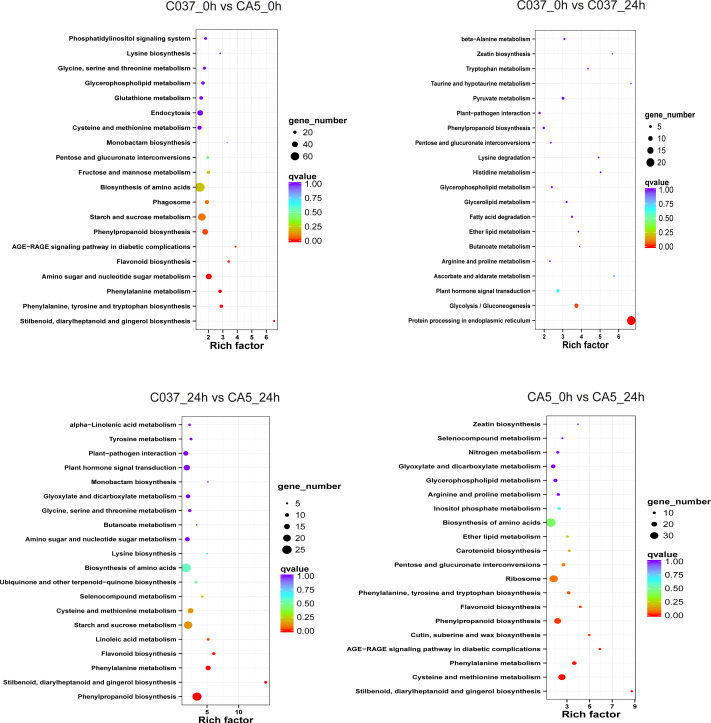
The top 20 enriched KEGG pathways corresponding to the DEGs in (A) C037_0 h *vs* C037_24 h, (B) C037_0 h *vs* CA5_0 h, (C) CA5_0 h *vs* CA5_24 h and (D) C037_24 h *vs* CA5_24h. The colour represents the Q-value, as shown in the figure. *Q*-values are the *P*-values corrected by multiple hypothesis testing, with a range of 0 to 1. The closer the Q-value is to zero, the more obvious the enrichment is. The size of each circle represents the number of DEGs in that pathway. The bigger the circle, the more DEGs.

The KEGG-based analysis further revealed that the number of DEGs in CA5 and C037 varied greatly in different physiological metabolic pathways under control and wind stress conditions (Fig. 6; Table S9). In C037_0 h *vs* C037_24 h, only pathways associated with protein processing in the endoplasmic reticulum (ko04141) and glycolysis/gluconeogenesis (ko00010) were significantly enriched under wind stress. In CA5_0 h *vs* CA5_24 h, eight KEGG pathways were significantly enriched under wind resistance. In C037_0 h *vs.* CA5_0 h, seven KEGG pathways were significantly enriched, and five metabolic pathways were significantly enriched in C037_24 h *vs.* CA5_24 h. Among them, stilbenoid, diallylheptanoid, and gingerol biosynthesis (ko00945), phenylpropanoid biosynthesis (ko00940), phenylalanine metabolism (ko00360), and flavonoid biosynthesis (ko00941) were involved in C037_0 h *vs.* CA5_0 h, CA5_0 h *vs.* CA5_24 h, C037_24 h *vs.* CA5_24 h. The four pathways may be related to the strong resistance of CA5 to wind stress. Moreover, phenylalanine biosynthesis (ko00940), phenylalanine metabolism (ko00360), and flavonoid biosynthesis (ko00941) pathways are all related to lignin biosynthesis. Therefore, it can be concluded that lignin content and various genes related to lignin synthesis are important factors, which cause variation in the levels of wind resistance in *Eucalyptus*.

### Differential expression analysis of cellulose synthesis genes

GO classification and enrichment analysis further highlighted the potential involvement of DEGs in cell biological process (GO: 0030244), cell microfibril organization (GO: 0010215), cell metabolic process (GO: 0030243), cell catalytic process (GO: 0030245), cell synthesis (UDP forming) activity (GO: 0016760) and cell synthesis activity (GO: 0016759), that is, six pathways related to cellulose synthesis upon wind stress. To better understand the involvement of cellulose synthesis genes in wind resistance in *E. camaldulensis*, the related DEGs were further analysed. Upon wind stress treatment, 26 genes related to cellulose biosynthesis were differentially expressed (Table 3, In the table, EC_newGene is the abbreviation for Eucalyptus_camaldulensis_newGene.), which were mainly assigned the following GO terms: cellulase synthase (UDP-forming) activity (GO: 0016760), cellulose biosynthetic process (GO: 0030244), and cellular microfibril organisation (GO: 0010215). No significant accumulation of cellulose synthesis genes was observed in C037 before and after wind stress treatment. Sixteen DEGs that were significantly enriched in CA5 were downregulated upon wind stress treatment. In total, 39 DEGs were significantly enriched in C037_0 h *vs.* CA5_0 h, of which 37 were upregulated and 2 were downregulated. Further, all the 11 DEGs in C037_24 h *vs.* CA5_24 h were upregulated. EC_newgene_41710, Gene003075, Gene008307, and Gene011799 were upregulated or downregulated in all groups except C037_0 h and C037_24 h. EC_newgene_68496, Gene008307, and Gene011799 were upregulated more than 10 times between C037 and CA5 before and after wind stress treatment. Therefore, Gene008307 and Gene011799 can be used as key candidate genes for wind resistance research in the future.

**Table 3:** Expression level of differentially expressed cellulose synthesis genes.

No.	Gene ID	FPKM value			
		C037_0 h	C037_24 h	CA5_0 h	CA5_24 h
1	Gene003075	28.398	19.048	571.015	205.706
2	Gene011799	27.944	11.366	749.046	201.560
3	EC_newGene_26230	1.275	0.617	0.000	0.064
4	EC_newGene_68240	0.968	0.196	11.482	1.461
5	EC_newGene_68495	1.756	4.296	20.599	3.072
6	EC_newGene_41710	18.063	7.740	513.351	153.859
7	Gene008709	3.280	1.832	6.065	1.907
8	EC_newGene_50309	0.000	0.000	2.760	0.593
9	EC_newGene_64334	7.896	9.077	17.147	19.484
10	Gene004875	29.646	19.262	63.598	33.001
11	EC_newGene_17715	0.000	4.626	23.453	13.142
12	EC_newGene_50014	2.151	1.946	92.386	12.453
13	EC_newGene_49931	23.290	22.420	51.884	49.156
14	EC_newGene_25516	0.494	0.752	4.325	3.113
15	Gene008307	25.093	10.995	460.534	137.980
16	EC_newGene_68242	0.167	0.595	5.320	0.720
17	EC_newGene_42873	0.511	3.246	8.986	1.118
18	EC_newGene_3993	1.449	1.637	9.361	1.980
19	EC_newGene_28579	35.848	41.349	72.731	71.383
20	Gene006185	0.532	0.395	6.326	2.016
21	EC_newGene_2305	1.580	1.113	57.704	7.346
22	EC_newGene_2296	2.299	1.481	2.275	0.981
23	EC_newGene_68520	2.494	3.384	10.237	11.544
24	EC_newGene_68496	0.000	4.027	3.222	0.203
25	EC_newGene_47118	0.547	0.249	19.032	2.536
26	Gene013116	58.964	59.971	133.828	93.281

### Differential expression analysis of lignin synthesis Genes

**Figure 7:**
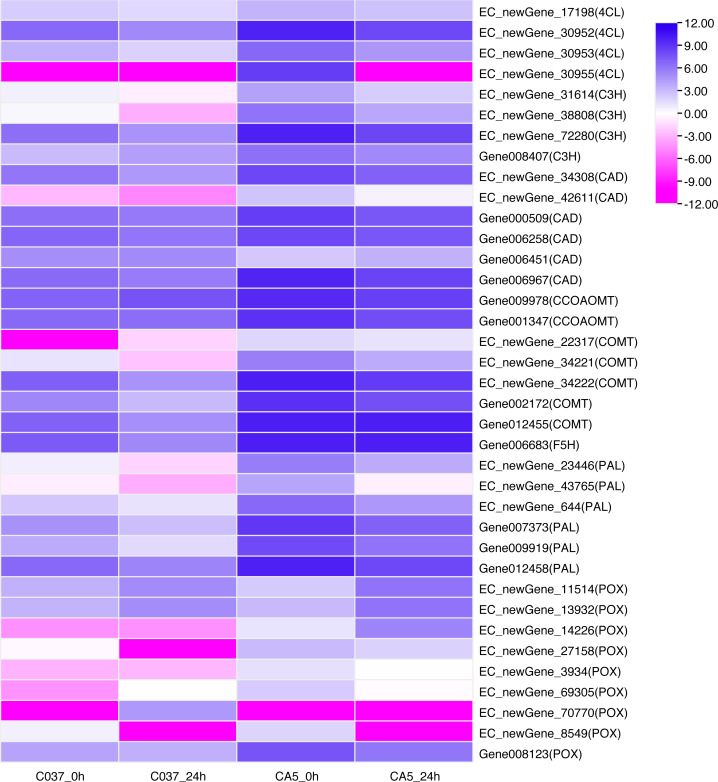
Heatmap of the differentially expressed genes of lignin monomer synthesis pathway based on KEGG phenylpropanoid biosynthetic pathways (KEGG Pathway map ID:mtr00940). The gene expression levels have been transformed by log2, (FPKM+1) and the values have been centred and scaled in row direction. *X*-axis, samples; *Y*-axis, gene names. Each colour represents the corresponding expression value, that is, the larger the value, the darker the colour, and the higher the expression.

KEGG-based pathways enrichment analysis revealed that some DEGs could be involved in lignin synthesis pathways. For instance, phenylpropanoid biosynthesis (ko00940) was observed Table S10) (Humphreys & Chapple, 2002). A total of 37 DEGs were annotated as phenylalanine ammonia-lyase (PAL), 4-Hydroxycinnamoyl-CoA ligase (4CL), cinnamate 3-hydroxylase(C3H), caffeic acid/5- hydroxyferulic acid O-methyltransferase (COMT), cinnamoyl-CoA reductase (CCR), cinnamyl-alcohol dehydrogenase (CAD), ferulate 5-hydroxylase (F5H), caffeoyl-CoA 3-O-methyltransferase (CCoAOMT), and peroxidase (POX). The expression levels of DEGs across the four samples were analysed (Fig. 7). The results showed that 8 out of 37 genes were present in the 4 groups. In C037_0 h *vs.* CA5_0 h, the downstream gene006451 (CAD) was down-regulated; however, the other 30 genes were upregulated, including EC_newGene_72280, gene002172, gene007373, gene009919, and gene012455, which were upregulated more than 10 times. In C037_24 h *vs.* CA5_24 h, all the other 18 key genes in the lignin monomer synthesis pathway were upregulated, except EC_newgene_70770 (POX) and Gene006451 (CAD). In CA5_0 h *vs.* CA5_24 h, the downstream regulatory genes EC_newgene_11514 (POX), EC_newgene_13932 (POX), and EC_NewGene_14226 (POX) were upregulated, while the other 20 key genes in the lignin monomer synthesis pathway were downregulated. EC_newGene_38808, EC_newGene_42611, EC_newGene_23446, EC_newGene_14226, and Gene012455 were upregulated more than 30 times across C037 and CA5 before and after wind stress treatment. EC_newGene_38808 and EC_newGene_14226, which were upregulated more than 45 times, could be considered key candidate genes involved in response to wind stress.

### TFs related to wind stress

**Figure 8:**
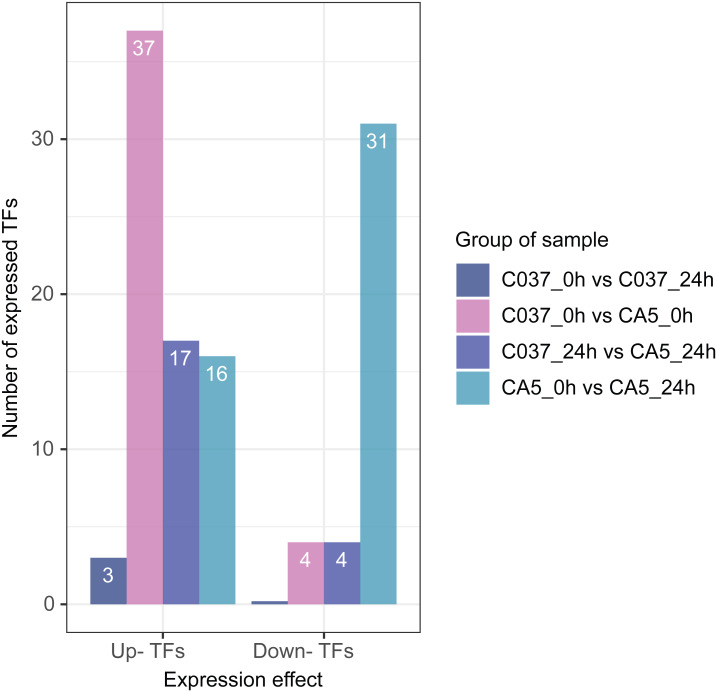
Differentially expressed TFs between four groups under wind stress.

Based on differential expression analysis and the available transcriptomic sequence information, TFs were predicted across the two genotypes of *E. maldulensis*. A total of 78 different TFs, which may be related to the regulation of cellulose and lignin synthesis were obtained (Fig. 8; Table S11). These differentially expressed TFs were primarily classified as 20 MYB families, 15 NAC families, 12 WRKY families, 12 bHLH families, and a small number of LIM, MADS, SBP, C3H, and other TFs. Four TFs were downregulated and 37 TFs were upregulated in C037_0 h *vs.* CA5_0 h; 17 TFs were upregulated and 4 TFs were downregulated in C037_24 h *vs.* CA5_24 h, and 11 TFs were up-regulated before and after C037 *vs.* CA5 wind stress treatment. Sixteen TFs were upregulated and 31 were downregulated after CA5 wind stress treatment, while only three TFs were upregulated after C037 wind stress treatment. This indicated that there was little difference in the expression levels of C037 before and after wind damage. These TFs are involved in the regulation of plant growth and development, morphogenesis, stress resistance, and other biological metabolic pathways.

**Figure 9:**
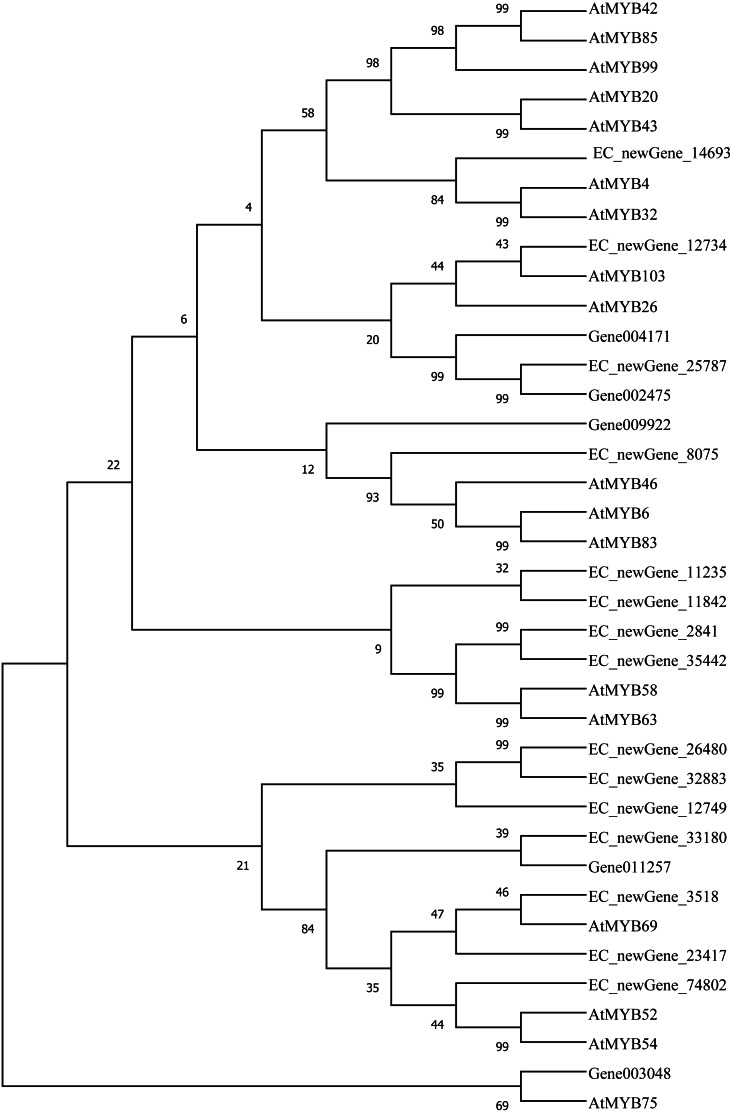
Phylogenetic analysis of differentially expressed MYB TFs. Using MEGA X software, the phylogenetic tree of 20 differentially expressed MYB TFs (including those TFs involved in the secondary cell wall synthesis in *Arabidopsis*) has been constructed based on the Neighbor-Joining method.

**Figure 10:**
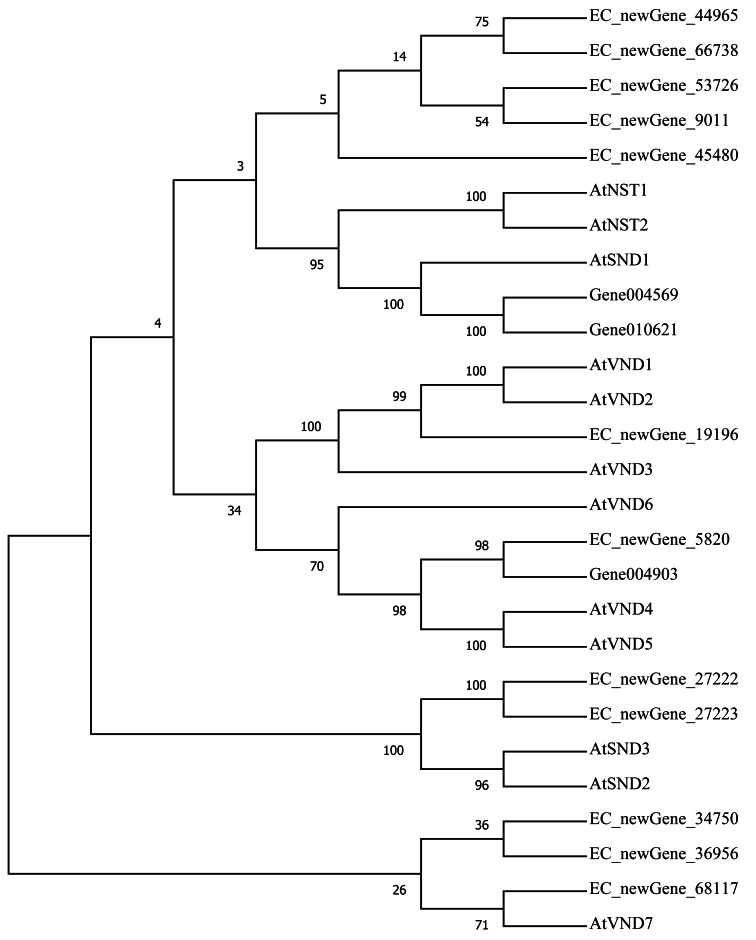
Phylogenetic analysis of differentially expressed NAC TFs. The phylogenetic tree of 15 differentially expressed NAC TFs (including those TFs involved in the secondary cell wall synthesis in *Arabidopsis*) has been constructed by using the Neighbour-Joining method in the MEGA X software.

Homologous evolution analysis was performed between the differentially expressed MYB and NAC TFs as well as the TFs involved in secondary wall synthesis in *Arabidopsis thaliana* (Figs. 9; 10). EC_newGene_8075 exhibited 93% similarity with ATMYB46, ATMYB6, ATMYB83, EC_newGene_2841, while EC_newGene_35442 showed 99% similarity with ATMYB58 and ATMYB63 (Fig. 7). Gene004569 and gene010621 exhibited a 100% similarity to ATSND1, likewise, EC_newGene_19196 was 100% similar to ATVND1, ATVND2, ATVND3. EC_newGene_5820 and Gene004903 exhibited 100% similarity with ATVND4 and ATVND5, while EC_newGene_27222 and EC_newGene_27223 were 100% similar to ATSND2 and ATSND3.

### Verification of sequencing results by qRT-PCR analysis

**Figure 11:**
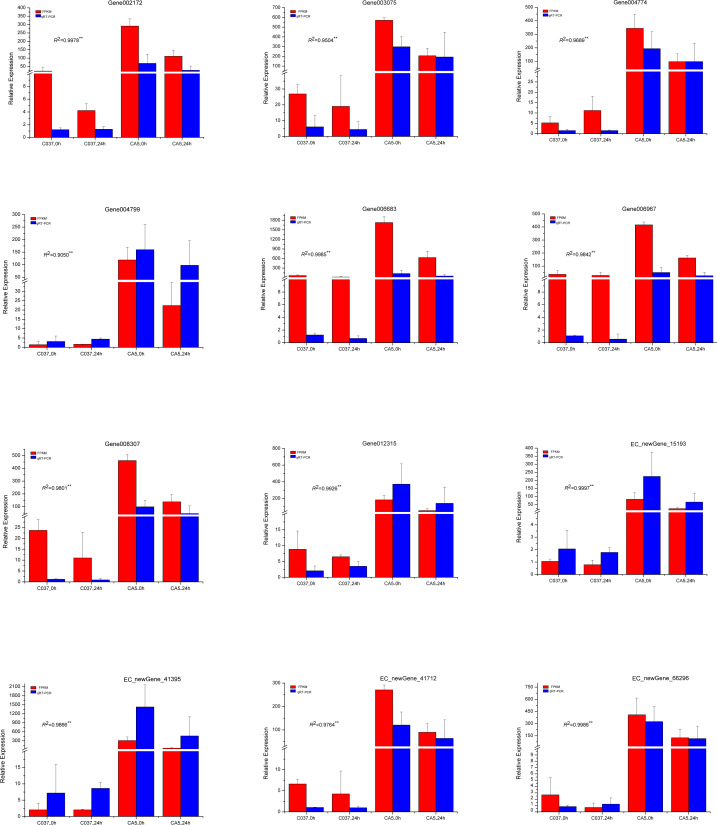
The expression levels of the 12 candidate unigenes related to cellulose biosynthesis and lignin biosynthesis as revealed by qRT-PCR and RNA-seq.

To verify the accuracy of the transcriptome sequencing results, the expression levels of 12 different genes related to cellulose and lignin synthesis were analysed by conducting qRT-PCR (Fig. 11). The qRT-PCR-based expression trends of these genes were consistent with the results of transcriptome sequencing, and the correlation coefficients ranged from 0.9050 to 0.9997, which are significant and indicate that the transcriptome sequencing data had high repeatability and quasi-certainty.

## Discussion

Transcriptomics in functional genomics is to study the expression and regulation mechanism of functional genes in specific tissues or cells at different developmental stages and in different states at the overall mRNA level, so as to clarify biological phenotypes and functions. Many stress-related gene expression, signal transduction and metabolite formation are involved in the process of plant tolerance to environmental stress. The application of RNA-seq technology can comprehensively and dynamically detect the expression changes of plant genes in various time and space under stress, mine functional genes and analyze the stress response regulation mechanism, which can provide a lot of useful information for understanding the complex mechanism of plant tolerance to abiotic stress and provide molecular genetic basis for cultivating stress resistant varieties. In this study, two contrasting varieties of *E. camaldulensis* CA5 and C037 were used as the materials. The traits related to wind resistance of lignin content, holocellulose content, *α*- cellulose content had high heritability and the genetic of upper and lower generations was strong. The number of DEGs in CA5 under wind stress was much higher than that in C037. A total of 726 DEGs specific to CA5 in response to wind stress were analysed. It was found that cell wall genes and phenylpropanoid biosynthesis play key roles in the response to wind stress. The results of the GO analysis and KEGG analysis showed that the genes related to wind stress were mainly related to secondary cell wall synthesis, that is, cellulose and lignin synthesis and related TFs. The results of this study are consistent with the research conclusions of crop lodging resistance.

### SNP sites based on RNA-sep

In the natural environment, the mutation frequency of plants is very low. The substances that can induce mutation are usually substances that have certain damage or stimulating effects on plants. Based on the transcriptome data of wind-resistant strain CA5 and non-wind resistant strain C037, SNP loci were screened, which confirmed that there were abundant SNP loci in *E. camaldulensis*. SNP loci located on genes were annotated. Based on these annotations, the selected wind resistance related genes or SNPs in the pathway may be directly related to wind resistance. However, not all SNPs related to traits are located on genes, and some SNPs located in non-coding regions also play an important role in biological phenotypes.

### Relationship between cellulose anabolism and wind stress

The cell wall of plants has a strong filamentous network structure, which can provide mechanical support for cells and the whole plant body; therefore, it can promote the maintenance of stem mechanical strength. The content of the cell wall can reflect the plant anti-lodging ability to a certain extent (Xiang et al., 2010; Hagiwara et al., 1999). In this study, 42 functional annotation genes were differentially expressed and were closely related to cellulose synthesis. The variation trend was consistent with the determination of cellulose and lignin content in the secondary wall, that is, the expression levels in CA5 were much higher than that in C037. This is consistent with the previous results corresponding to the lodging resistance of crops. The increase in cellulose content in wheat straw significantly improves the mechanical strength of the stem and enhances the compressive capacity of the wheat stem (Fan et al., 2012). A significant positive correlation was observed between the breaking resistance of the rice basal stem and the contents of lignin and cellulose (Yang et al., 2009). High cellulose content is beneficial for improving the stem strength of soybeans and enhancing their lodging resistance (Deng et al., 2016). Therefore, the cellulose content in the cell wall and its mechanical properties may have a significant impact on the wind resistance of trees. In this study, EC_newgene_68496, Gene008307, and Gene011799 were upregulated more than 10 times between C037 and CA5 genotypes before and after wind stress treatment, which can be considered as key candidate genes for wind resistance research in the future.

### Relationship between lignin anabolism and wind resistance

Lignin can improve the strength of the cell wall and the mechanical strength of the stem. The existence of lignin not only enhances the defence ability of plants against biological stress, but also has great significance in waterlogging resistance, cold resistance, lodging resistance, and other abiotic stresses (Whetten & Sederoff, 1995). The results of a study on lodging resistance of crops have revealed that lignin can improve the cell wall strength and enhance the mechanical strength of the stem, and the content of lignin is closely related to stem rigidity (Turner & Somerville, 1997). Changing the composition of lignin does not affect the growth and development of plants (Vanholme et al., 2012); however, increasing its content can significantly enhance the compressive and lodging resistance in stems. Moreover, the strength of the lodging resistance is proportional to the extent of mechanical strength (Jones, Ennos & Turner, 2001; Baucher et al., 2003). For example, the presence of lignin improves the lodging resistance of wheat, rice, rape, corn, and other important food crops. The lignin content of varieties with strong lodging resistance is significantly higher than that of varieties without lodging resistance (Li et al., 2015; Buranov & Mazza, 2008; Kim & Dale, 2004; Ookawa & Ishihara, 1992). Some studies have shown that the expression levels of PAL, 4CL, C4H, and C3H in the phenylalanine secondary metabolism pathway not only affect the synthesis and content of lignin but also affect the biosynthesis of other secondary metabolites (Baucher et al., 2003). The expression levels of F5H, COMT, and CCOAOMT have a greater impact on the composition of lignin monomers, while CAD and CCR significantly affect the composition of lignin monomers (Boerjan, Ralph & Baucher, 2003). In this study, we identified several DEGs in the lignin monomer synthesis pathway in the phenylpropanoid biosynthetic pathway (ko00940); the expression levels of PAL, C3H, 4CL, COMT, CCoAOMT, CAD, F5H, and POX in CA5 were higher than those in C037, which further indicates that the expression of lignin synthesis genes in wind-resistant strains was higher than that in C037. These DEGs are likely to be involved in the regulation of cellulose and lignin content/component changes in *E. camaldulensis*, which provides a theoretical basis for screening the major genes affecting wind resistance in *E. camaldulensis*. Based on their expression profile, EC_newGene_38808 and EC_newGene_14226 can be used as key candidate genes for wind resistance research in the future, as these were found to be upregulated more than 45 times in CA5.

### The relationship between TFs and wind resistance

Transcription factors play an important role in the regulatory network of plant stress resistance (Singh et al., 2002). Some transcription factors participate in the regulation of a variety of stress responses. The main transcription factor families involved in plant stress resistance include WRKY family, NAC family, AP2 / ERF family, bZIP family, MYB family, et. Many TFs are involved in the regulation of wind damage in *E. camaldulensis*. Among the 78 differential TFs that may be related to the regulation of cellulose and lignin synthesis, the MYB and NAC families were the most abundant ones. ATMYB83 and ATMYB46 are nonspecific synthesis activators, which directly target genes of SND1 and node genes regulating secondary wall formation in *Arabidopsis thaliana*. They regulate lignin synthesis in addition to the overall synthesis of the secondary cell wall including cellulose and hemicellulose. When overexpressed in *A. thaliana*, it can activate the biosynthetic pathways of cellulose, xylan, and lignin, and activate the expression of promoter genes related to secondary wall synthesis (Zhong, Richardson & Ye, 2007; Mccarthy, Zhong & Ye, 2009). EC_newGene_8075 has 93% similarity with ATMYB83 and ATMYB46; therefore, we can speculate that EC_newGene_8075 may have similar functions. The specific expression of ATMYB58 and ATMYB63 in cells with thickened secondary wall material deposition is regulated by the transcription switch factor ATSND1 and its downstream TF ATMYB46. They activate gene expression by binding to AC elements on promoters of key enzymes in the lignin monomer synthesis pathway, such as 4CL and F5H. Hence, they are important regulators of lignin biosynthesis (Zhou et al., 2009; Hartmann, Sagasser & Mehrtens, 2005). EC_newGene_2841 and EC_newGene_35442 have 99% similarity with ATMYB58 and ATMYB63; therefore, it can be speculated that these two TFs have similar functions. Arabidopsis NAC domain transcription factor SND1 is a key transcription switch that regulates the synthesis of a secondary wall in fibres, which can activate the expression of the ATMYB46 gene, induce the highly upregulated expression of secondary wall-related genes, and promote the deposition of cellulose, xylan, lignin, and secondary wall in cells (Hartmann, Sagasser & Mehrtens, 2005; Zhong et al., 2011). Studies have shown that ATSND2 and ATSND3 are secondary TFs that are directly regulated by ATSND1 (Zhong et al., 2008) and are involved in the regulation of secondary cell wall thickening. It can be inferred that Gene004569, Gene010621, EC_newGene_27222, and EC_newGene_27223 may have similar functions. Among the TFs which are highly similar to MYB and NAC in *Arabidopsis*, EC_newGene_2841(MYB) and EC_newGene_5820(NAC) were upregulated more than nine times in CA5. The two TFs may account for the significant differences in the wind stress resistance between the two genotypes; therefore, these can be used as candidate genes for further functional verification analysis through overexpression experiments.

## Conclusions

Significant differences of DEG in two extreme wind-resistant genotypes can reveal that the wind resistance of Eucalyptus depended largely on the differential gene expression. Plant cell walls are highly complex and dynamic structures that, in addition to providing mechanical support and supporting growth, need to respond to a variety of environmental and developmental cues and play an important role in resisting biological and abiotic stresses. The results suggest that there were abundant SNP loci in *E. camaldulensis* and cell wall biogenesis key genes of cellulose and lignin biosynthesis pathways and related TFs play key roles in wind stress response. Eucalyptus with strong wind resistance had a high content of cellulose and lignin, it can be inferred that genes and TFs related to cellulose and lignin synthesis may participate in the regulation of Eucalyptus wind stress. Gene008307 and Gene011799 of cellulose biosynthesis, EC_newGene_38808 (C3H), and EC_newGene_14226 (POX) of lignin biosynthesis, EC_newGene_2841 (MYB), and EC_newGene_5820 (NAC) of transcription factors can be used as key candidate genes for wind resistance research in the future. This study provides insights into the wind resistance mechanism and molecular breeding in forests. Identification of the exact mechanisms through which cell wall biosynthesis; key genes of cellulose and lignin biosynthesis pathways and related TFs confers wind tolerance will be important in order to inform the development of wind tolerant trees. The combination of RNA-seq and DNA sequencing can detect SNP, RNA editing and expression quantitative trait loci. In particular, the genetic mechanism of complex traits can be obtained by correlation analysis of genotype data and gene expression changes. Transcriptomics is associated with proteomics, metabolomics and other omics for integrated analysis, which can be better applied to the identification of key plant stress resistance genes. Our next work will combine transcriptomics with other omics to study the wind resistance mechanism of forest trees more systematically and deeply from many aspects.

##  Supplemental Information

10.7717/peerj.12954/supp-1 Supplemental Information 1 Primers used in this study and amplification products

10.7717/peerj.12954/supp-2 Supplemental Information 2 Reaction system of qRT-PCR

10.7717/peerj.12954/supp-3 Supplemental Information 3 Sequencing data quality statistics

10.7717/peerj.12954/supp-4 Supplemental Information 4 The number of annotated genes identified in searches of eight databases

10.7717/peerj.12954/supp-5 Supplemental Information 5 Statistics of SNP in C037 and CA5

10.7717/peerj.12954/supp-6 Supplemental Information 6 Annotated DEGs of different groups

10.7717/peerj.12954/supp-7 Supplemental Information 7 Specific genes in CA5

10.7717/peerj.12954/supp-8 Supplemental Information 8 The numbers of DEGs in different KEGG pathways

10.7717/peerj.12954/supp-9 Supplemental Information 9 Gene expression trends in the phenylpropanoid biosynthetic pathway (ko00940) based on KEGG(C037_0 h *vs* CA5_0h)

10.7717/peerj.12954/supp-10 Supplemental Information 10 Volcano plots representing differentially expressed genes

10.7717/peerj.12954/supp-11 Supplemental Information 11 Expression levels of differentially expressed transcription factors

## Additional Information and Declarations

### DNA Deposition

The following information was supplied regarding the deposition of DNA sequences: The RNA-Seq reads are available at Sequence Read Archive (SRA): SRP326456.

### Data Availability

The following information was supplied regarding data availability: The RNA-Seq reads are available at Sequence Read Archive (SRA): SRP326456.

## References

[ref-1] Arnold RJ, Xie YJ, Midgley SJ, Luo JZ, Chen XF (2013). Emergence and rise of eucalypt veneer production in China. International Forestry Review.

[ref-2] Baucher M, Halpin C, Petit-Conil M, Boerjan W (2003). Lignin: genetic engineering and impact on pulping. Critical Reviews in Biochemistry and Molecular Biology.

[ref-3] Benjamini Y, Hochberg Y (1995). Controlling the false discovery rate: a practical and powerful approach to multiple testing. Journal of the Toyal Statistical Society.

[ref-4] Boerjan W, Ralph J, Baucher M (2003). Lignin biosynthesis. Annual Review of Plant Biology.

[ref-5] Braam J, Sistrunk ML, Polisensky DH, Purugganan MM, Antosiewicz DM, Campbell P, Johnson KA (1997). Plant responses to environmental stress: regulation and functions of the Arabidopsis TCH genes. Planta.

[ref-6] Browning BL (1967). Methods of wood chemistry.

[ref-7] Buranov AU, Mazza G (2008). Lignin in straw of herbaceous crops. Industrial Crops and Products.

[ref-8] Chehab EW, Eich E, Braam J (2009). Thigmomorphogenesis: a complex plant response to mechano-stimulation. Journal of Experimental Botany.

[ref-9] Coomes DA, Šafka D, Shepherd J, Dalponte M, Holdaway R (2018). Airborne laser scanning of natural forests in New Zealand reveals the influences of wind on forest carbon. Forest Ecosystems.

[ref-10] Défossez P, Yang M, Bonnefond JM, Garrigou D, Trichet P, Danijon F (2015). Tree resistance to wind: the effects of soil conditions on tree stability. Congrés Franc˛ais de Mécanique.

[ref-11] Deng YC, Liu WG, Yuan XQ, Yuan J, Zou JL, Du JB, Yang WY (2016). Relationship between cellulose synthesis metabolism and lodging resistance in intercropping soybean at seedling stage. Chinese Journal of Applied Ecology.

[ref-12] Duperat M, Gardiner B, Ruel JC (2021). Testing an individual tree wind damage risk model in a naturally regenerated balsam first stand: potential impact of thinning on the level of risk. Forestry.

[ref-13] Dupont S, Défossez P, Bonnefond JM, Irvine MR (2018). How stand tree motion impacts wind dynamics during windstorms. Agricultural and Forest Meteorology.

[ref-14] Edzang E, Badel E, Pitti RM, Gril J, Bruno M (2020). Characterization of the thigmomorphogenetic response of rubber tree clone (hevea brasiliensis): impact on wind breakage strength. https://hal.archives-ouvertes.fr/hal-03042580.

[ref-15] Ennos AR (1997). Wind as an ecological factor. Trends in Ecology & Evolution.

[ref-16] Espírito-Santo FDB, Gloor M, Keller M, Malhi Y, Saatchi S, Nelson B, Junior RCO, Pereira C, Lloyd J, Frolking S, Palace M, Shimabukuro YE, Duarte V, Mendoza AM, López-González G, Baker TR, Feldpausch TR, Brienen RJW, Asner GP, Boyd DS, Phillips OL (2014). Size and frequency of natural forest disturbances and the Amazon forest carbon balance. Nature Communications.

[ref-17] Fan WX, Hou YX, Feng SW, Zhu SW, Ru ZG (2012). Study on cellulose and lodging resistance of wheat straw. Journal of Henan Agricultural Sciences.

[ref-18] Fredericksen TS, Hedden RL, Williams SA (1993). Testing loblolly pine wind firmness with simulated wind stress. Canadian Journal of Forest Research.

[ref-19] Gardiner B, Berry P, Moulia B (2016). Wind impacts on plant growth, mechanics and damage. Plant Science.

[ref-20] Gardiner B, Byrne K, Hale S, Kamimura K, Mitchell SJ, Peltola H, Ruel JC (2008). A review of mechanistic modeling of wind damage risk to forests. Forestry.

[ref-21] Gilbert HJ (2010). The biochemistry and structural biology of plant cell wall deconstruction. Plant Physiology.

[ref-22] Gregow H, Puranen U, Venäläinen A, Peltola H, Kellomäki S, Schultz D (2008). Temporal spatial occurrence of strong winds large snowfall amounts in Finland during 1961-2000. Silva Fennica.

[ref-23] Hagiwara M, Izusawa H, Inoue N, Matano T (1999). Varietal differences of shoot growth characters related to lodging in tartary buckwheat. Fagopyrum.

[ref-24] Hartmann U, Sagasser M, Mehrtens F (2005). Differential combinatorial interactions of cis-acting elements recognized by R2R3-MYB, BZIP, and BHLH factors control light-responsive and tissue-specific activation of phenylpropanoid biosynthesis genes. Plant Molecular Biology.

[ref-25] Huang H, Chen DL, Chang Y, Hu WH, Wu CS, Gu Y (2014). Studies on variences of the lodging- resistant ability and the mechanism in different maize varieties. Journal of Nanjing Agricultural University.

[ref-26] Humphreys JM, Chapple C (2002). Rewriting the lignin roadmap. Current Opinion in Plant Biology.

[ref-27] Huntley SK, Ellis D, Gilbert M, Chapple C, Mansfield SD (2003). Significant increases in pulping efficiency in C4H-F5H-transformed poplars: improved chemical savings and reduced environmental toxins. Agricultural Food Chemstry.

[ref-28] Imbert D, Portecop J (2008). Hurricane disturbance and forest resillience: assessing structural *vs*. functional changes in a Caribbean dry forest. Forest Ecology and Management.

[ref-29] Jackson T, Shenkin A, Kalyan B, Zionts J, Calders K, Origo N, Disney M, Burt A, Raumonen P, Malhi Y (2019). A new architectural perspective on wind damage in a natural forest. Frontiers in Forests and Global Change.

[ref-30] James KR, Haritos N, Ades PK (2006). Mechanical stability of trees under dynamic loads. American Journal of Botany.

[ref-31] Jones L, Ennos AR, Turner SR (2001). Cloning and characterization of *irregular xylem4* (*irx4*): A severely lignin-deficient mutant of *Arabidopsis*. The Plant Journal.

[ref-32] Kim SB, Dale E (2004). Global potential bioethanol production from wasted crops and crop residues. Biomass & Bioenergy.

[ref-33] Lewis NG, Yamamoto E (1990). Lignin: occurrence, biogenesis and biodegradation. Annual Review of Plant Physiology and Plant Molecular Biology.

[ref-34] Li FC, Zhang ML, Guo K, Hu Z, Zhang R, Feng YQ, Yi XY, Zou WH, Wang LQ, Wu CY, Tian JS, Lu TG, Xie GS, Peng LC (2015). High-level hemicellulosic arabinose predominately affects lignocellulose crystallinity for genetically enhancing both plant lodging resistance and biomass enzymatic digestibility in rice mutants. Plant Biotechnology Journal.

[ref-35] Liu TX, Guan CY (2008). Grey relational analysis between lodging index and biochemistry components of stem, agronomic characteristics in rapeseed *(Brassica napus* L.). Chinese Journal Oil Crop Sciences.

[ref-36] Livak KJ, Schmittgen TD (2001). Analysis of relative gene expression data using Real-Time Quantitative PCR and the 2^−ΔΔ^^CT^ method. Methods.

[ref-37] Luo JZ, Arnold R, Lu WH, Lin Y (2014). Genetic variation in *Eucalyptus camaldulensis* and *E. tereticornis* for early growth and susceptibility to the gall wasp *Leptocybe invasa* in china. Euphytica.

[ref-38] Luo JZ, Xie YJ, Cao JG, Lu WH, Ren SQ (2009). Genetic variation in 2-year Eucalypt hybrids’ growth and typhoon resistance. Acta Pratacult Urae Sinica.

[ref-39] Mao XZ, Cai T, Olyarchuk JG, Wei LP (2005). Automated genome annotation and pathway identification using the KEGG Orthology (KO) as a controlled vocabulary. Bioinformatics.

[ref-40] Mccarthy RL, Zhong RQ, Ye ZH (2009). MYB83 is a direct target of SND1 and acts redundantly with MYB46 in the regulation of secondary cell wall biosynthesis in *Arabidopsis*. Plant and Cell Physiology.

[ref-41] Nicoll BC, Easton EP, Milner AD, Walker C, Coutts MP (1995). Wind stability factors in tree selection: distribution of biomass within root systems of Sitka spruce clones.

[ref-42] Ookawa T, Ishihara K (1992). Varietal difference of physical characteristics of the culm related to lodging resistance in paddy rice. Japanese Journal of Crop Science.

[ref-43] Ookawa T, Ishihara KV (1993). Varietal difference of cell wall components affecting the bending stress of the culm in relating to lodging resistance in paddy rice. Japanese Journal of Crop Science.

[ref-44] Peltola H, Gardiner BA, Kellomäki S, Kolström T, Lässig R, Moore JR (2000). Wind and other abiotic risks to forests. Forest Ecology and Management.

[ref-45] Sani L, Lisci R, Moschi M, Sarri D, Rimediotti M, Vieri M, Tofanelli S (2012). Preliminary experiments and verification of controlled pulling tests for tree stability assessments in Mediterranean urban areas. Plant Molecular Biology.

[ref-46] Shang XH, Zhang PJ, Xie YJ, Luo JZ, Li C, Wu ZH (2017). Wind resistance correlated to growth and wood properties of 50 *Eucalyptus camaldulensis* provenance families. Journal of Zhejiang a & F University.

[ref-47] Singh K, Foley RC, Oñate-Sánchez L (2002). Transcription factors in plant defense stress responses. Current Opinion in Plant Biology.

[ref-48] Telewski FW, Pruyn ML (1998). Thigmomorphogenesis: a dose response to flexing in ulmus americana seedlings. Tree Physiology.

[ref-49] Thompson DL (1963). Stalk strength of corn as measured by crushing strength and rind thickness. Crop Sciece.

[ref-50] Trapnell C, Pachter L, Salzberg SL (2005). TopHat: discovering splice junctions with RNA-Seq. Bioinformatics.

[ref-51] Trapnell C, Williams BA, Pertea G, Mortazavi A, Kwan G, Baren MJV, Salzberg SL, Wold BJ, Pachter L (2010). Transcript assembly and quantification by RNA-Seq reveals unannotated transcripts and isoform switching during cell differentiation. Nature Biotechnologh.

[ref-52] Tripathi SC, Sayre KD (2003). Growth and morphology of wheat (*Triticum aestivum* L.) culms and their association with lodging: effects of genotypes, N levels and ethephon. Field Crops Research.

[ref-53] Turner SR, Somerville CR (1997). Collapsed xylem phenotype of Arabidopsis identifies mutants deficient in cellulose deposition in the secondary cell wall. The Plant Cell.

[ref-54] Vanholme R, Morreel K, Darrah C, Oyarce P, Grabber JH, Ralph J, Boerjan W (2012). Metabolic engineering of novel lignin in biomass crops. New Phytologist.

[ref-55] Welton FA (1928). Lodging in oats and wheat. Botan Gazatte.

[ref-56] Whetten R, Sederoff R (1995). Lignin biosynthesis. The Plant Cell.

[ref-57] Wu CT, Huang HS, Gao XS, Zhang WS, Li WG (2012). Comparative study on wind- resistance of 21 *Hevea brasiliensis* clones. Journal of Fujian College of Forestry.

[ref-58] Wu ZH, Li TH, Zhang HL, Xie YJ (2010). Studies on growth and wind-resistance traits of *Casuarina* and *Acacia* stands from coastal protection forest. Acta Pratacult Urae Sinica.

[ref-59] Xiang DB, Guo K, Lei T, Yu XB, Luo QM, Yang WY (2010). Effects of phosphorus and potassium on stem characteristics and lodging resistance of relay cropping soybean. Chinese Journal of Oil Crop Sciences.

[ref-60] Xie YJ, Arnold RJ, Wu ZH, Chen SF, Du AP, Luo JZ (2017). Advances in eucalypt research in China. Frontiers of Agricultural Science and Engineering.

[ref-61] Xu X, Cheng YW, Jiang HL, Li XP, Liu YH (2017). Research progress of the effects of wind speed change on grassland ecosystem. Acta Ecologica Sinica.

[ref-62] Xu XY, Wang MH, Zhong CL, Zhang HX (2014). Wood properties and anti-typhoon performance in selected trees. Journal of Zhejiang A & F University.

[ref-63] Yang SM, Xie L, Zheng SL, Li J, Yuan JC (2009). Effects of nitrogen rate and transplanting density on physical and chemical characteristics and lodging resistance of culms in hybrid rice. Acta Agronomica Sinica.

[ref-64] Young MD, Wakefield MJ, Smyth GK, Oshlack A (2010). Gene ontology analysis for RNA-seq: accounting for selection bias. Genome Biology.

[ref-65] Zanuncio AJV, Carvalho AG, Carneiro ACO, Colodette JL, Rocha MFV (2019). Chemical and energetic characterization of *Eucalyptus grandis*×*Eucalyptus urophylla* clones subject to wind damage. Revista áRvore.

[ref-66] Zanuncio AJV, Carvalho AG, Carneiro ACO, Valenzuela P, Gacitua W, Leite FP, Colodette JL (2017). Characterization of eucalyptus clones subject to wind damage. Pesquisa Agropecuária Brasileira.

[ref-67] Zhong R, Lee C, McCarthy RL, Reeves CK, Jones Ye ZH (2011). Transcriptional activation of secondary wall biosynthesis by rice and maize NAC and MYB transcription factors. Plant Cell Physiology.

[ref-68] Zhong R, Lee C, Zhou J, McCarthy RL, Ye ZH (2008). A battery of transcription factors involved in the regulation of secondary cell wall biosynthesis in Arabidopsis. The Plant Cell.

[ref-69] Zhong R, Richardson EA, Ye ZH (2007). The MYB46 transcription factor is a direct target of SND1 and regulates secondary wall biosynthesis in *Arabidopsis*. The Plant Cell.

[ref-70] Zhou J, Lee C, Zhong R, Ye ZH (2009). MYB58 and MYB63 are transcriptional activators of the lignin biosynthetic pathway during secondary cell wall formation in *Arabidopsis*. The Plant Cell.

[ref-71] Zuber MS, Grogan CO (1961). A new technique for measuring stalk strength in corn. Crop Science.

[ref-72] Zubizarreta-Gerendiain A, Pukkala A, Peltola H (2016). Effects of wind damage on the optimal management of boreal forests under current and changing climatic conditions. Canadian Journal of Forest Research.

