# Supplementary material for: Comparative transcriptomics analysis of contrasting varieties of Eucalyptus camaldulensis reveals wind resistance genes
Source: PeerJ. 2022 Feb 24;10:e12954. doi: 10.7717/peerj.12954 (PMC8882336; doi:10.7717/peerj.12954)
Supplement: Supplemental Information 3 [file peerj-10-12954-s003.docx]

**Table** **S3: Sequencing data quality statistics**

| Sanple Name | Clean Reads Numble | Clean bases Numble | Clean Reads Q20 (%) | Clean Reads Q30 (%) | GC Number(%) |
| --- | --- | --- | --- | --- | --- |
| C037-0h-1 | 20849140 | 6244644418 | 98.01 | 94.86 | 51.45 |
| C037-0h-2 | 24526394 | 7342646438 | 97.67 | 94.06 | 51.77 |
| C037-24h-1 | 20321837 | 6085178700 | 97.95 | 94.75 | 52.05 |
| C037-24h-2 | 25779996 | 7717953118 | 98.12 | 95.18 | 51.77 |
| CA5-0h-1 | 19697418 | 5893116176 | 97.87 | 94.44 | 51.01 |
| CA5-0h-2 | 22722036 | 6799516612 | 98.09 | 94.98 | 51.68 |
| CA5-0h-3 | 20988986 | 6278297726 | 97.83 | 94.26 | 51.34 |
| CA5-24h-1 | 28879406 | 8639188918 | 98.07 | 94.88 | 51.87 |
| CA5-24h-2 | 22052110 | 6601358458 | 98.08 | 94.88 | 51.23 |
| CA5-24h-3 | 25935940 | 7754692790 | 98.07 | 94.88 | 51.87 |
