# Supplementary material for: Comparative transcriptomics analysis of contrasting varieties of Eucalyptus camaldulensis reveals wind resistance genes
Source: PeerJ. 2022 Feb 24;10:e12954. doi: 10.7717/peerj.12954 (PMC8882336; doi:10.7717/peerj.12954)
Supplement: Supplemental Information 4 [file peerj-10-12954-s004.docx]

**Table S4: The number of annotated genes identified in searches of eight databases**

| #Anno_Database | Annotated_Number |
| --- | --- |
| COG_Annotation | 7,620 |
| GO_Annotation | 19,217 |
| KEGG_Annotation | 9,898 |
| KOG_Annotation | 13,885 |
| Pfam Annotation | 15,138 |
| Swissprot_Annotation | 17,951 |
| eggNOG | 22,867 |
| nr_Annotation | 26,523 |
| All_Annotated | 26,588 |
