# Supplementary material for: Comparative transcriptomics analysis of contrasting varieties of Eucalyptus camaldulensis reveals wind resistance genes
Source: PeerJ. 2022 Feb 24;10:e12954. doi: 10.7717/peerj.12954 (PMC8882336; doi:10.7717/peerj.12954)
Supplement: Supplemental Information 5 [file peerj-10-12954-s005.docx]

Table S5 Statistics of SNP in C037 and CA5

| Types | | C037-1 | | C037-2 | | C037-3 | | CA5-1 | | CA5-2 | | CA5-3 | |
| --- | --- | --- | --- | --- | --- | --- | --- | --- | --- | --- | --- | --- | --- |
|  |  | Numbers | Percentage  (%) | Numbers | Percentage  (%) | Numbers | Percentage  (%) | Numbers | Percentage  (%) | Numbers | Percentage  (%) | Numbers | Percentage  (%) |
| Tansitions | C/T | 42268 | 48.57 | 20947 | 32.32 | 17910 | 31.15 | 21143 | 31.39 | 20005 | 31.46 | 19228 | 31.48 |
|  | A/G | 23292 | 26.77 | 22875 | 35.30 | 20727 | 36.05 | 23777 | 35.30 | 22484 | 35.36 | 21869 | 35.80 |
|  | Sum | 65560 | 75.34 | 43822 | 67.62 | 38637 | 67.20 | 44920 | 66.69 | 42489 | 66.82 | 41097 | 67.27 |
| Tranversions | A/C | 5665 | 6.51 | 5480 | 8.46 | 4977 | 8.66 | 5919 | 8.79 | 5565 | 8.75 | 5299 | 8.67 |
|  | G/T | 5207 | 5.98 | 5015 | 7.74 | 4533 | 7.88 | 5295 | 7.86 | 4998 | 7.86 | 4822 | 7.89 |
|  | C/G | 5285 | 6.07 | 5299 | 8.18 | 4843 | 8.42 | 5616 | 8.34 | 5320 | 8.37 | 5063 | 8.29 |
|  | A/T | 5301 | 6.09 | 5189 | 8.01 | 4506 | 7.84 | 5611 | 8.33 | 5212 | 8.20 | 4808 | 7.87 |
|  | Sum | 21458 | 24.66 | 20983 | 32.38 | 18859 | 32.80 | 22441 | 33.31 | 21095 | 33.18 | 19992 | 32.73 |
|  | Total | 87018 |  | 64805 |  | 57496 |  | 67361 |  | 63584 |  | 61089 |  |
| Heterozygosity | |  | 39.03 |  | 37.01 |  | 45.55 |  | 42.50 |  | 42.94 |  | 44.04 |
