# Supplementary figures and images for: Comparative transcriptomics analysis of contrasting varieties of Eucalyptus camaldulensis reveals wind resistance genes
Source: PeerJ. 2022 Feb 24;10:e12954. doi: 10.7717/peerj.12954 (PMC8882336; doi:10.7717/peerj.12954)

## PHENYLPROPANOID BIOSYNTHESIS

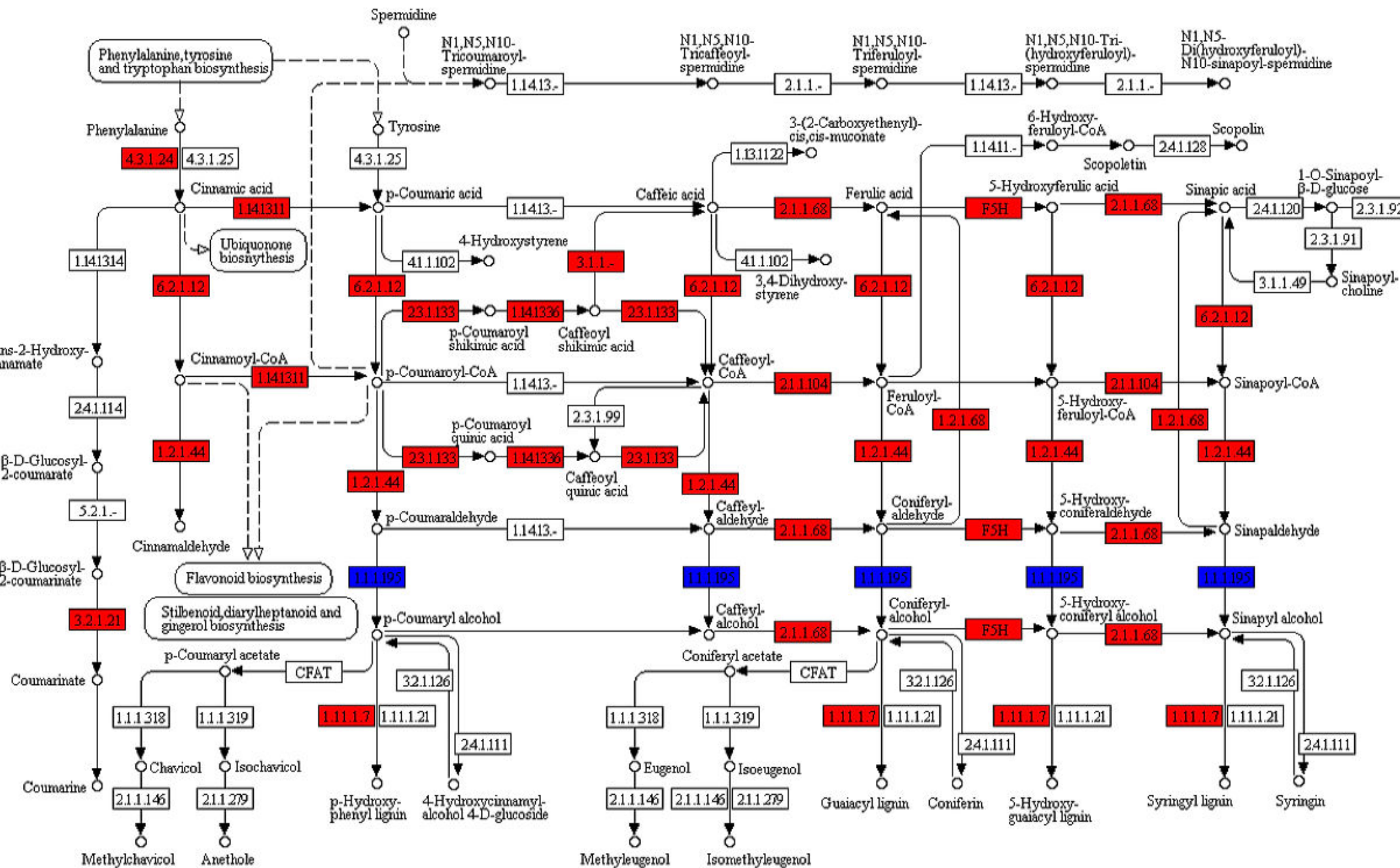

Supplement: Supplemental Information 9 [file peerj-10-12954-s009.pdf]

C037\_0h VS C037\_24h

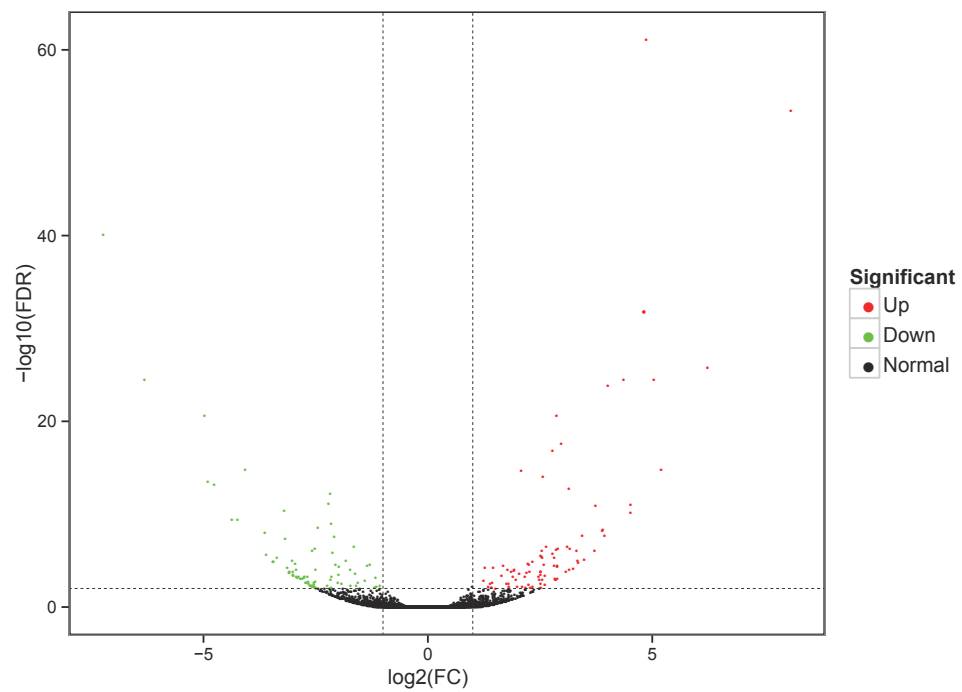

C037\_0h VS CA5\_0h

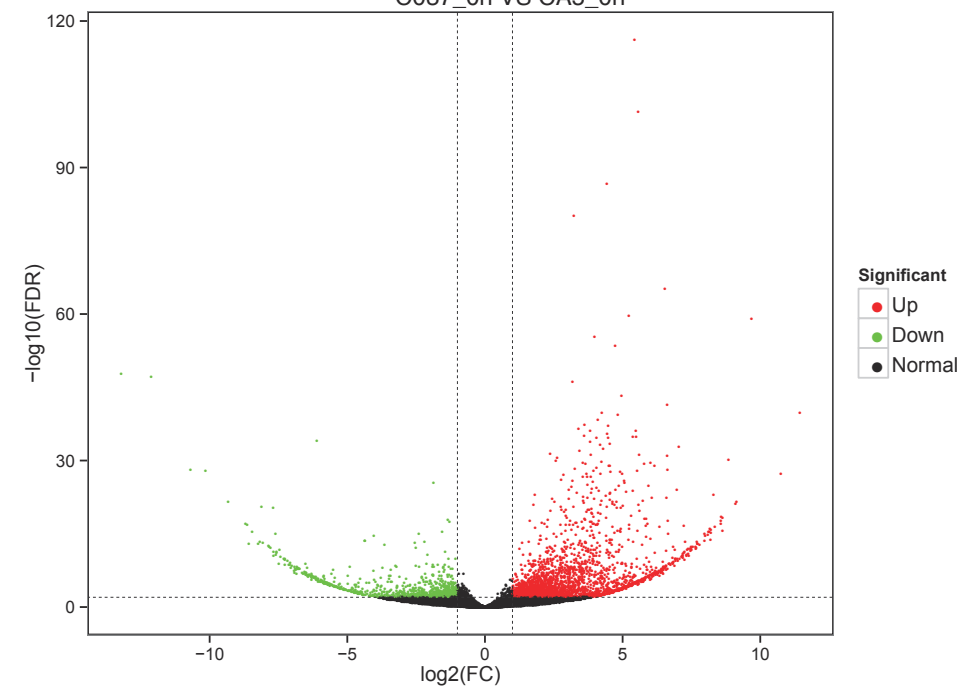

C037\_24h VS CA5\_24h

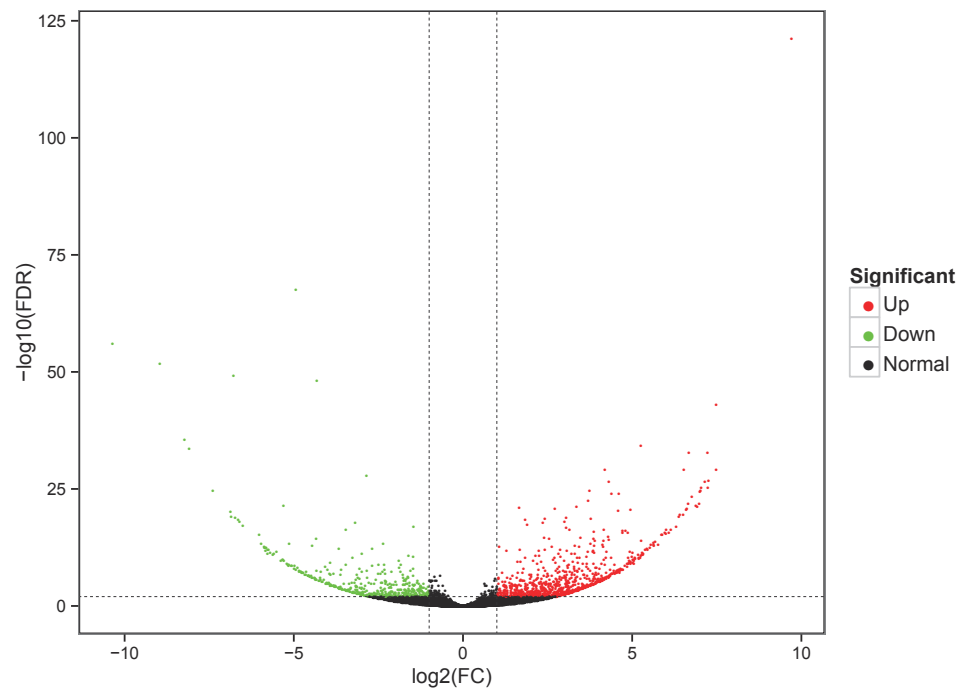

CA5\_0h VS CA5\_24h

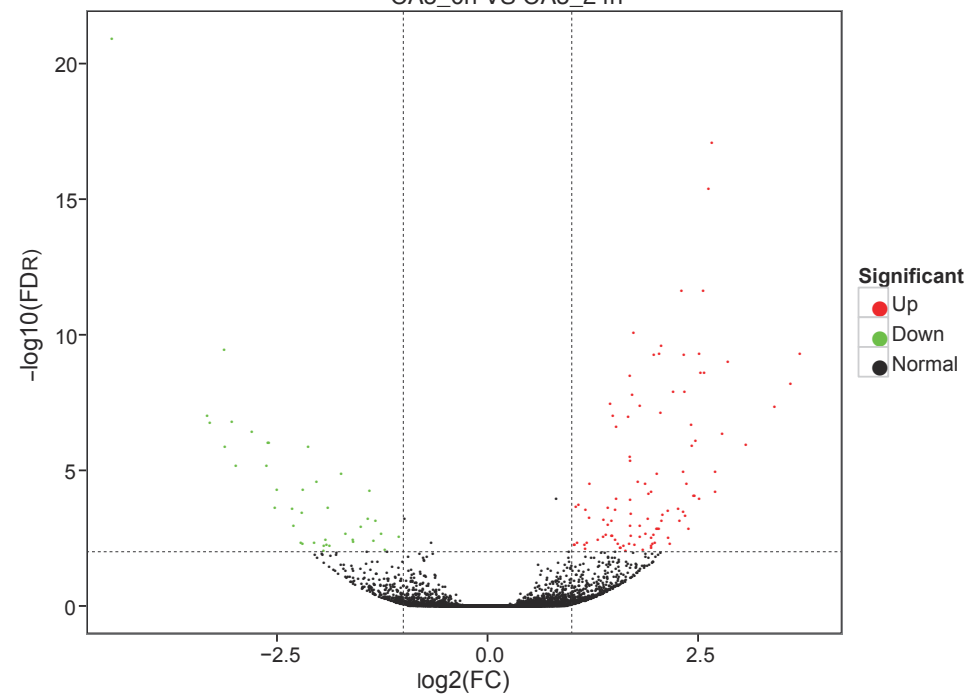

Supplement: Supplemental Information 10 [file peerj-10-12954-s010.pdf]
